# Assessment of Ophthalmology Teaching and its Impact on the Choice of Future Specialties Among Medical Students of Jazan University

**DOI:** 10.7759/cureus.49134

**Published:** 2023-11-20

**Authors:** Ismail Abuallut, Eman Hurissi, Bandar M Abuageelah, Mona Alfaifi, Alshomokh Hakami, Alanoud Qadri, Afnan Hakami, Saleh Ghulaysi

**Affiliations:** 1 Department of Surgery, Ophthalmology Division, Jazan University, Jazan, SAU; 2 Department of Surgery, Ophthalmology Division, Prince Mohammed Bin Naser Hospital, Jazan, SAU; 3 Department of Medicine and Surgery, Batterjee Medical College, Aseer, SAU; 4 Department of Medicine, Emergency Division, General Jazan Hospital, Jazan, SAU; 5 Department of Pediatrics, General Jazan Hospital, Jazan, SAU; 6 Department of Pharmacy, Maternity & Children's Hospital Bisha, Bisha, SAU; 7 Medical School, Jazan University, Jazan, SAU

**Keywords:** jazan, specialty, education, curriculum, undergraduate, ophthalmology

## Abstract

Background: Ophthalmology is essential for primary and specialty care physicians, as eye complaints are common, accounting for a sizable proportion of general practice consultations and emergency department visits. Fundamental ophthalmology knowledge is also relevant to other specialty fields. Thus, medical schools must provide effective undergraduate curricula to teach students about salient points, visual examination skills, emergency recognition, and referral indications. The International Council of Ophthalmology (ICO) has set guidelines that medical students should imbibe to become proficient in ophthalmology. However, there have been no recent investigations evaluating undergraduate ophthalmology education at Jazan University's Faculty of Medicine. Therefore, this study aimed to compare the curriculum at Jazan University to the ICO's requirements for undergraduate medical education.

Methods: An observational cross-sectional study was conducted with both male and female student participants enrolled in an ophthalmology course at Jazan University's Faculty of Medicine. Following IRB approval, the questionnaire was distributed on social media to assess if Jazan University's undergraduate ophthalmology curriculum complies with ICO standards.

Results: The study included a diverse sample of 322 participants, predominantly consisting of female students (n=178, 55.3%). The participants' ages ranged from 22 to 36 years, with the majority falling within the 24-25 year age range (n=173, 53.7%). Regarding academic performance, (n=117, 36.3%) of participants had a GPA of less than 4, while 66 (20.5%) had a GPA between 4.76 and 5.00. Among the respondents, 31 (9.6%) indicated having no exposure to ophthalmology, while 117 (36.3%) felt they had insufficient exposure. A considerable percentage of participants expressed competence in various areas, such as obtaining ocular history (n=113, 35.1%), testing visual acuity (n=201, 62.4%), and examining extraocular motility (n=201, 62.4%). In total, 98 participants (30.4%) expressed an interest in ophthalmology, while the majority (n=224, 69.6%) were not interested.

Conclusion: Essential improvements include increasing hands-on clinical experience, small-group learning, exposure across academic years, and early mentorship. Developing competency-based curricula aligned with ICO guidelines could significantly strengthen education. Better training quality and exposure will equip students with the necessary skills, boost confidence, and potentially expand the ophthalmology workforce.

## Introduction

Primary and specialty care physicians must have a good understanding of the core principles and techniques of ophthalmology. This is especially relevant given that eye complaints are among the most common symptom complaints of patients presenting to health care practitioners [1,2]; ophthalmology accounts for a sizable proportion of primary care consultations, accounting for 3-19% of general practice consultations [3,4]. The proportion of ophthalmic cases presenting to accident and emergency departments may be far higher than seen in general practice. As a result, fundamental ophthalmic abilities are critical for primary care practitioners, a profession that a large majority of medical students enter [3].

Additionally, ophthalmology has relevance for other specialty fields like endocrinology, neurology, pediatrics, rheumatology, cardiology, and nephrology [5]. As a result, fundamental ophthalmology expertise is essential to care for patients with specific neurologic, endocrine, rheumatic, and infectious conditions. Thus, medical schools must use effective undergraduate curricula that enable students to become proficient in the fundamentals of ophthalmology before beginning employment. This includes acquiring essential knowledge, specifically in conducting ophthalmic history-taking, building proficiency in specific visual examination abilities, developing the skill to identify ophthalmic emergencies, and understanding the criteria for referring patients in the field of ophthalmology [6]. Furthermore, having this essential knowledge and abilities in ophthalmology is critical for medical students when deciding on a future profession. The method by which medical subjects are taught and the duration of exposure to medical subjects impact the decision to pursue a specific medical career [7].

The International Council of Ophthalmology (ICO) created comprehensive guidelines that outline the core competencies and recommend using particular teaching techniques, including lectures, clinical demonstrations, illustrative case studies, and evidence-based medicine instruction that medical students should achieve upon completing medical school to become primary care physicians capable of handling ophthalmic pathologies [6]. Unfortunately, in recent years, a detailed official investigation has not been conducted regarding undergraduate ophthalmology education at Jazan University's Faculty of Medicine. As a result, we compared the self-reported efficacy of the undergraduate ophthalmology curriculum to the requirements for undergraduate medical education set by the ICO.

## Materials and methods

An observational cross-sectional study was conducted among students enrolled in an ophthalmology course, a three-week course for fifth-year students. The course offers three credit hours with different types of instruction, such as lectures, tutorials, bedside teaching, and case-based learning at Jazan University's Faculty of Medicine. The study method involves random distribution of a questionnaire via social media to students who took and completed Jazan University's ophthalmology course. The total number of students enrolled in the five-year ophthalmology course at Jazan University was 1188, with 590 men and 598 women. A sample size of 291 respondents was determined using Raosoft software (Raosoft Inc., Seattle, WA, USA, www.raosoft.com) based on an estimated number of students of 1188, a 95% confidence level, and a 5% error margin. The snowball convenience sampling method was utilized in this cross-sectional study. Following ethical approval from Jazan University's ethical approval committee (reference number REC-45/02/733, date 03 September 2023), the researchers distributed the survey link via various social media platforms (Twitter, WhatsApp, and Telegram) to their key contacts (i.e. students who took and completed the Jazan University's Ophthalmology course); they were then requested to pass on the survey to their own contacts, and so on.

Based on a review of the literature [1], a validated, self-administered questionnaire was obtained. The questionnaire included sections to evaluate ophthalmology exposure, education quality, and teaching methods in this institution. It also assessed student understanding of the significance of the ophthalmology course, their knowledge and competency after the ophthalmology course in medical school, and the impacts of ophthalmology courses on choosing ophthalmology as a specialty. Additionally, a section inquiring about the participants' demographics was included at the beginning of the questionnaire. Descriptive statistics, including means, standard deviations, medians, and interquartile ranges, were used to describe the characteristics of the studied sample. Quantitative data were analyzed using t-tests, and the association of qualitative variables was examined using chi-square tests. A p-value less than 0.05 was considered statistically significant. The privacy of participants was ensured, as all questionnaires were anonymous, and no identification data was collected. Data were stored in an electronic encrypted format and were only accessed by the investigators listed in the application.

## Results

The study included a diverse sample of 322 participants, predominantly consisting of females (n=178, 55.3%). The participants' ages ranged from 22 to 36 years, with the majority falling within the 24-25 age range (n=173, 53.7%). Regarding academic performance, (n=117, 36.3%) of participants had a GPA less than 4, while (n=66, 20.5%) had a GPA between 4.76 and 5.00. In terms of academic years, the largest group was in their internship year (n=129, 40.1%), followed by those in their sixth academic year (n=89, 27.6%). These demographic factors provide a comprehensive overview of the participants involved in the study, ensuring a varied representation for the subsequent analyses (Table 1).

**Table 1:** The demographic factors of the included participants. Standard deviation (SD), minimum (Min) and maximum (Max) values of age.

Parameter	Category	N (%)
Gender	Male	144 (44.7%)
	Female	178 (55.3%)
Age	22-23	87 (27.0%)
	24-25	173 (53.7%)
	26-30	53 (16.5%)
	> 30	9 (2.8%)
Mean Age (SD, Min-Max)	24.64 (2.12, 22-36)	
GPA	Less than 4	117 (36.3%)
	4.00-4.50	77 (23.9%)
	4.51-4.75	62 (19.3%)
	4.76-5.00	66 (20.5%)
Academic Year	5th	42 (13.0%)
	6th	89 (27.6%)
	Internship	129 (40.1%)
	Graduate	62 (19.3%)

**Figure 1:**
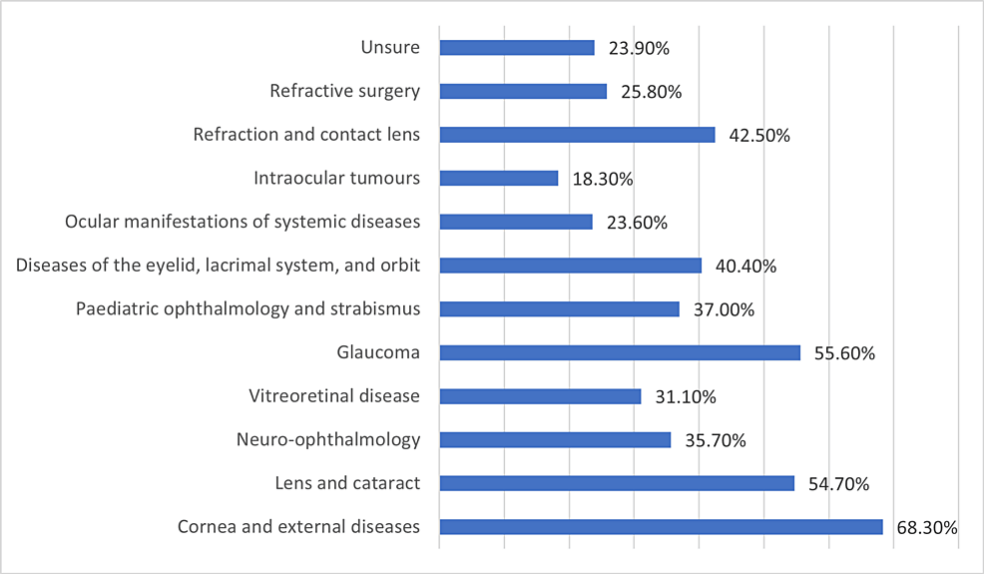
Which areas in ophthalmology were you exposed to during medical school?

Table 2 provides an assessment of the level of exposure to ophthalmology received during medical school, as reported by the participants. Among the respondents, (n=31, 9.6%) indicated having no exposure to ophthalmology, while (n=117, 36.3%) felt they had insufficient exposure. On the other hand, (n=138, 42.9%) believed they had received an appropriate level of exposure, and a smaller proportion (n=18, 5.6%) considered the exposure to be excessive. A similar pattern emerged when participants were asked to rate the quality of ophthalmology education in their medical school: four (1.2%) reported no ophthalmology training, while 30 (9.3%) and 52 (16.1%) assessed the education as very poor and poor, respectively. Conversely, 125 (38.8%) perceived the quality as average, and 67 (20.8%) and 44 (13.7%) rated it as very good and excellent, respectively. The most common teaching methods employed were theoretical lectures (n=283, 87.9%) and self-directed learning (n=85, 26.4%), while smaller proportions involved small-group discussions (n=63, 19.6%) and clinical settings such as the emergency room, operating room, and clinics (n=54, 16.8%). In terms of practical exposure, 138 of the participants (42.9%) reported having taken ophthalmic history or performed ophthalmic examinations, while 184 (57.1%) had not. When encountering ophthalmic patients, the majority had no contact (n=202, 62.7%), with a minority (n=56, 17.4%) encountering them in ophthalmology clinics. The areas of ophthalmology most commonly encountered during medical school included cornea and external diseases (n=220, 68.3%), lens and cataract (n=176, 54.7%), and glaucoma (n=179, 55.6%), among others (Figure 1).

**Table 2:** The measurement of the level of exposure ophthalmology received in medical school.

Parameter	Category	N (%)
In your opinion, how much exposure to ophthalmology did you receive in medical school?	No exposure	31 (9.6%)
	Too little exposure	117 (36.3%)
	Just the right amount	138 (42.9%)
	Too much exposure	18 (5.6%)
	Unsure	18 (5.6%)
In your opinion, how would you rate the quality of ophthalmology education in your medical school?	I did not receive any ophthalmology training	4 (1.2%)
	Very poor	30 (9.3%)
	Poor	52 (16.1%)
	Average	125 (38.8%)
	Very good	67 (20.8%)
	Excellent	44 (13.7%)
The most common method/s used for teaching	Theoretical lectures	283 (87.9%)
	Small-group discussions	63 (19.6%)
	Clinical (ER,OR, clinics)	54 (16.8%)
	Self-directed learning	85 (26.4%)
Have you been exposed, took ophthalmic history or ophthalmic examination of patients during the ophthalmology course in your medical school?	No	184 (57.1%)
	Yes	138 (42.9%)
In your medical college, which of the following locations have you most encountered ophthalmic patients?	No contact with ophthalmic patients	202 (62.7%)
	Ophthalmology clinic	56 (17.4%)
	Emergency department	10 (3.1%)
	Operating room	14 (4.3%)
	Family medicine practice	33 (10.2%)
	Other	7 (2.2%)

Table 3 presents the assessment of student awareness regarding the importance of the ophthalmology course. When asked about the relevance of learning ophthalmology for their particular residency program, 157 participants (48.8%) responded positively, while 120 (37.3%) expressed a neutral stance, and 45 (14.0%) believed it was not important. Similarly, a majority of participants recognized the importance of learning how to conduct a complete ocular history (n=229, 71.1%) and the slit-lamp examination (n=225, 69.9%). Additionally, a significant proportion (n=264, 82.0%) acknowledged the significance of knowing the indications for referral to ophthalmology services.

**Table 3:** Assessment of the student awareness regarding the importance of the ophthalmology course.

Parameter	No	Neutral	Yes
	N (%)	N (%)	N (%)
Do you believe that learning about ophthalmology was important for your particular residency program?	45 (14.0%)	120 (37.3%)	157 (48.8%)
Do you believe that learning how to conduct a complete ocular history is important?	38 (11.8%)	55 (17.1%)	229 (71.1%)
Do you believe that learning about slit-lamp examination is important?	25 (7.8%)	72 (22.4%)	225 (69.9%)
Do you believe that knowing the indication for referral to ophthalmology services is important?	22 (6.8%)	36 (11.2%)	264 (82.0%)

In Table 4, the students' self-assessed knowledge and competency after the ophthalmology course are presented. A considerable percentage of participants expressed competence in various areas, such as obtaining ocular history (n=113, 35.1%), testing visual acuity (n=201, 62.4%), and examining extraocular motility (n=201, 62.4%). However, a higher percentage felt incompetent in areas such as assessing intraocular pressure (n=217, 67.4%) and anterior chamber depth (n=236, 73.3%). Similarly, a significant proportion expressed a lack of confidence in performing specific procedures, including slit-lamp examination (n=216, 67.1%) and corneal examination with fluorescein dye (n=237, 73.6%).

**Table 4:** Assessment of the student's knowledge and competency after the ophthalmology course in medical school.

Parameter	Incompetent	Competent
	N (%)	N (%)
How competent do you feel obtaining ocular history?	209 (64.9%)	113 (35.1%)
How competent do you feel testing the visual acuity?	121 (37.6%)	201 (62.4%)
How competent do you feel doing visual field examination?	104 (32.3%)	218 (67.7%)
How competent do you feel in examining Extra ocular motility?	121 (37.6%)	201 (62.4%)
How competent do you feel in doing Pupillary light reflex assessment?	131 (40.7%)	191 (59.3%)
How competent do you feel in doing pupillary dilation, and Fundoscopy with direct ophthalmoscope?	207 (64.3%)	115 (35.7%)
How competent do you feel in doing Slit-lamp examination?	216 (67.1%)	106 (32.9%)
How competent do you feel in doing corneal examination with fluorescein dye?	237 (73.6%)	85 (26.4%)
How competent do you feel in the assessment of intraocular pressure?	217 (67.4%)	105 (32.6%)
How competent do you feel in the assessment of anterior chamber depth?	236 (73.3%)	86 (26.7%)
How competent do you feel in knowing and understanding the Indications for referral to ophthalmology services?	180 (55.9%)	142 (44.1%)

Table 5 provides insights into the impacts of ophthalmology courses on students' interest in pursuing ophthalmology as a specialty. Among the participants, 98 (30.4%) expressed an interest in ophthalmology; the majority (n=224, 69.6%) were not interested. However, when asked if their ophthalmology course in medical school contributed to their interest in the specialty 89 (27.6%) responded positively. Similarly, 89 participants (27.6%) considered applying for an ophthalmology residency program. When examining the strongest factors influencing their decision to apply for an ophthalmology residency program, the summer training at the hospital was cited by a majority of the participants (n=121, 40.1%), followed closely by an ophthalmology course at medical school (n=109, 36.1%) and mentorship (n=72, 23.8%).

**Table 5:** Impacts of ophthalmology courses on choosing Ophthalmology as a specialty.

Parameter	Category	N (%)
Are you interested in ophthalmology specialty?	No	224 (69.6%)
	Yes	98 (30.4%)
Did your ophthalmology course in medical school contribute to your interest in ophthalmology specialty?	No	233 (72.4%)
	Yes	89 (27.6%)
Do you consider applying for an ophthalmology residency program?	No	233 (72.4%)
	Yes	89 (27.6%)
What is the strongest factor that contributes to your decision to apply for an ophthalmology residency program?	Ophthalmology course at medical school	109 (36.1%)
	Summer training at the hospital	121 (40.1%)
	Mentor	72(23.8%)

Furthermore, Table 6 provides a breakdown of the participants' interest in ophthalmology based on gender, age, GPA, academic year, exposure to ophthalmology in medical school, and the quality of ophthalmology education. The data shows that gender distribution did not significantly influence interest in ophthalmology. However, age, GPA, academic year, exposure to ophthalmology, and the quality of ophthalmology education were found to have statistically significant associations with interest in ophthalmology as a specialty. Specifically, younger individuals, those with higher GPAs, students in lower academic years, and those who had more exposure to ophthalmology or received higher-quality ophthalmology education were more likely to express an interest in ophthalmology.

**Table 6:** The factors affecting the interest of the students regarding the ophthalmology specialty *Statistically significant (p < 0.05).

Parameter	Category	Are you interested in the ophthalmology specialty?		p-value
		No	Yes	
		N (%)	N (%)	
Gender	Male	103 (71.5%)	41 (28.5%)	0.491
	Female	121 (68.0%)	57 (32.0%)	
Age	22-23	56 (64.4%)	31 (35.6%)	0.003*
	24-25	131 (75.7%)	42 (24.3%)	
	26-30	35 (66.0%)	18 (34.0%)	
	> 30	2 (22.2%)	7 (77.8%)	
GPA	Less than 4	91 (77.8%)	26 (22.2%)	0.000*
	4.00-4.50	58 (75.3%)	19 (24.7%)	
	4.51-4.75	43 (69.4%)	19 (30.6%)	
	4.76-5.00	32 (48.5%)	34 (51.5%)	
Academic Year	5th	27 (64.3%)	15 (35.7%)	0.000*
	6th	49 (55.1%)	40 (44.9%)	
	Internship	105 (81.4%)	24 (18.6%)	
	Graduate	43 (69.4%)	19 (30.6%)	
In your opinion, how much exposure to ophthalmology did you receive in medical school?	No exposure	12 (38.7%)	19 (61.3%)	0.001*
	Too little exposure	88 (75.2%)	29 (24.8%)	
	Just the right amount	98 (71.0%)	40 (29.0%)	
	Too much exposure	11 (61.1%)	7 (38.9%)	
	Unsure	15 (83.3%)	3 (16.7%)	
In your opinion, how would you rate the quality of ophthalmology education in your medical school?	I did not receive any ophthalmology training	3 (75.0%)	1 (25.0%)	0.000*
	Very poor	18 (60.0%)	12 (40.0%)	
	Poor	44 (84.6%)	8 (15.4%)	
	Average	95 (76.0%)	30 (24.0%)	
	Very good	49 (73.1%)	18 (26.9%)	
	Excellent	15 (34.1%)	29 (65.9%)	

## Discussion

This study involved a diverse sample of 322 medical students, providing a comprehensive representation of perspectives on ophthalmology education. The findings reveal several important insights. Notably, a considerable proportion of students reported insufficient exposure and poor quality of ophthalmology training in medical school. Over a third believed their exposure was inadequate, and around a quarter rated the quality as poor or very poor. These findings are consistent with those of previous studies, which have also shown that ophthalmology education in medical school is often inadequate. This highlights major deficiencies in ophthalmology education that need to be addressed. A previous study conducted in the United States showed that there is a decline in ophthalmology’s role in medical student curricula [8]. Moreover, another study conducted in Saudi Arabia showed that the ophthalmology curricula did not adequately cover 35.8% of the basic portion, 61.3% of the clinical portion, 26.4% of common diseases, and 39.6% of common emergencies. More than 80% of the participating residents expressed that the ophthalmology course needs to be improved, regarding effectiveness most of them considered that the course is not helpful in familiarizing future general practitioners with ophthalmic emergencies and diseases, overall ophthalmology curricula required a significant improvement [9]. In another study conducted in Saudi Arabia, out of the 145 invitees, 120 (82.8%) responded. Around 50% percent of respondents reported overall satisfaction with the program while adequate clinical exposure was reported in most subspecialties except refraction and low vision rehabilitation with inadequate exposure reported by 55.8% and 95.8%, respectively [10]. Furthermore, an online survey was distributed to eligible first-year residents who had graduated from Canadian medical schools, the survey was categorized by basic statistics and demographic variables. The data obtained from 386 of a total of 1425 participants showed that the majority (64%) of them had almost no exposure to ophthalmology in medical school, and (76.2%) stated that they had one week or even less overall exposure to ophthalmology; while competency was obtained in some ICO clinical skills, such as visual acuity assessment and pupillary reflexes, the other clinical skills such as fundoscopy, slit-lamp examination and assessment of intraocular pressure were not achieved. Overall, the study showed that the undergraduate ophthalmology training in Canadian medical schools contained gaps in specific key areas; ensuring the competency of a medical student in ophthalmic skills and knowledge of a nationally standardized curriculum could be helpful for future clinical practice [11]. In the United Kingdom, a lack of proper undergraduate education has led to significant deficits in essential ophthalmology knowledge and skills, especially among those who worked in emergency departments [12], and 63.9% of senior house officers lacked confidence in their ability to manage eye emergencies [13]. Non-specialist physicians should be able to perform basic ophthalmologic examinations, including direct ophthalmoscopy, especially when access to an ophthalmologist is unavailable. Specifically, the findings of direct ophthalmoscopy may indicate debilitating systemic changes, such as the detection of papilledema (indicating accelerated hypertension or lesions occupying the cerebral space) as well as cytomegalovirus retinitis and endocarditis, which require additional invasive investigations [14,15]. Identification of common avoidable eye diseases and early referral of patients to eye care specialists is another key part of healthcare that can lower the prevalence of community ophthalmological disorders [16]. Therefore, there is an urgent need to make substantial modifications to the undergraduate medical curriculum in order to boost patient safety and future doctors' competence.

In line with ICO guidelines, most participants recognized the value of competencies such as taking an ocular history and knowing when to refer patients to ophthalmology services. However, self-assessed proficiency was lower in practical skills such as slit-lamp examination and tonometry. This underscores the need for more hands-on clinical training to develop competency. Exposure through lectures was far more common than small group discussions or clinical settings. Integrating more interactive and experiential learning approaches could enhance proficiency. A study conducted by Alselaimy and Alalawi (2021) among 31 different private and public medical schools in various regions of Saudi Arabia with 317 students participants showed that most of the Saudi medical schools that followed the ICO guidelines are much more competent in numerous ophthalmology basic skills. To ensure that future general practitioners are capable of dealing with different ophthalmic patients, structured national guidelines must be set in order to establish gold-standard eyesight health programs in Saudi Arabia [17]. Another cross-sectional examination of United Kingdom (UK) medical schools revealed major disparities in organization and teaching methods, as well as curricula that did not match ICO recommendations [18]. In addition, in Canada, just 35.7% of schools have instituted a mandated ophthalmology rotation, and the duration of each was often less than two weeks [19]. More than half of the students at the New York University School of Medicine felt uneasy diagnosing eye crises, and they lacked confidence while measuring visual acuity and using a direct ophthalmoscope [20].

In addition, significant competence gaps existed in areas such as slit-lamp examination, tonometry, and anterior segment assessment. These are concerning given their importance for detecting key ocular conditions, as these skills are essential for detecting key ocular conditions [21]. For example, insufficient tonometry skills can lead to inaccurate intraocular pressure measurements and missed diagnoses of glaucoma [22]. Low anterior segment competency also can make it difficult to diagnose cataracts and corneal disease [23]. These deficiencies can lead to missed diagnoses and delayed referrals if students do not receive adequate clinical training. Building proficiency in slit-lamp examination is particularly crucial, yet only 32.9% felt competent. Without proper training on slit-lamp usage and anterior segment evaluation, examination quality suffers [24]. This could result in missed diagnoses and suboptimal patient care. Closing these competency gaps through increased hands-on learning should be a priority. Simulation labs and standardized patient encounters focused on slit-lamp usage, tonometry, and anterior segment exams would provide vital practice. Implementing competency benchmarks aligned with ICO guidelines would help track progress. Addressing these training deficiencies is imperative for equipping students to provide quality, comprehensive eye care.

In addition, the present study highlights that only 30.4% of students expressed interest in pursuing ophthalmology. In a previous study conducted in the US, the authors reported that 15% of the students were interested in ophthalmology at medical school entry [25]. However, the ophthalmology course influenced over a quarter of respondents to consider the specialty. This indicates that high-quality medical school training can play a pivotal role in exposing students to ophthalmology and potentially inspiring them to help address shortages [26,27]. Clinical exposure such as summer hospital training and mentorship were also key factors, suggesting that hands-on learning motivates interest. Tailoring instruction and mentorship to students' levels could further enhance engagement. A key finding was that higher exposure to ophthalmology and better quality of education were associated with greater interest in pursuing ophthalmology. This reinforces that sufficient clinical exposure and high-quality training are essential to inspiring students to consider specializing. Tailoring education to students' level of knowledge is also vital, as interest declined in later academic years. Optimizing ophthalmology education can thus help address shortages in the specialty.

Several recommended enhancements could be considered to enhance student proficiency. First, expanding the length of ophthalmology lectures for undergraduates may increase their knowledge and interest in the field. Li et al. demonstrated that introducing additional ophthalmology lectures to third-year undergraduates greatly boosted their knowledge and influenced their motivation to teach [28]. Second, the contribution of the entire teaching team enables the integration of fundamental scientific knowledge and clinical practice for small student groups [29]. Thirdly, training techniques, such as didactic lectures, case presentations, and PowerPoint presentations, may have considerable benefits with variable efficacy outcomes [30,31]. The time of educational training is also an important factor. Four-year ophthalmology programs are likely to be more effective than one- or two-year programs, as indicated by previous research [20,32,33]. Lastly, offering comments throughout instructional sessions would be quite beneficial for fostering examination skills. Therefore, such multi-optional approaches should be considered in future reforms of the undergraduate ophthalmology course at medical schools, in order to improve the teaching process and the ophthalmology curriculum and practice of students. There were some limitations to this study. First, we only evaluated eye skills rather than particular knowledge learned from ophthalmology curricula. The ICO suggestions for the knowledge composition of a perfect ophthalmology module should be considered. Second, the ICO identified some clinical ocular skills that were overlooked: pupillary size measurement and interpretation, penlight examination of the anterior segment including upper lid eversion, ability to remove superficial corneal or conjunctival foreign bodies, and evaluating the red reflex. Furthermore, because the data was gathered by self-reporting, the findings may have limitations, resulting in an accurate categorization bias if some participants are required to comprehend the questions.

## Conclusions

Overall, this study demonstrates that current ophthalmology training in medical school is suboptimal. Essential improvements include increasing hands-on clinical experience, small-group learning, exposure across academic years, and early mentorship. Developing competency-based curricula aligned with ICO guidelines could significantly strengthen education. Better training quality and exposure will equip students with necessary skills, boost confidence, and potentially expand the ophthalmology workforce. Further studies could also explore longer-term outcomes of enriched ophthalmology education. Implementing these recommendations can help produce skilled graduates prepared to provide quality eye care.
